# Towards a Dynamic Interaction Network of Life to unify and expand the evolutionary theory

**DOI:** 10.1186/s12915-018-0531-6

**Published:** 2018-05-29

**Authors:** Eric Bapteste, Philippe Huneman

**Affiliations:** 1 Sorbonne Universités, UPMC Université Paris 06, Institut de Biologie Paris-Seine (IBPS), F-75005 Paris, France; 2 CNRS, UMR7138, Institut de Biologie Paris-Seine, F-75005 Paris, France; 3 Institut d’Histoire et de Philosophie des Sciences et des Techniques (CNRS / Paris I Sorbonne), F-75006 Paris, France

**Keywords:** Evolutionary biology, Interactions, Theoretical biology, Tree of Life, Web of Life

## Abstract

The classic Darwinian theory and the Synthetic evolutionary theory and their linear models, while invaluable to study the origins and evolution of species, are not primarily designed to model the evolution of organisations, typically that of ecosystems, nor that of processes. How could evolutionary theory better explain the evolution of biological complexity and diversity? Inclusive network-based analyses of dynamic systems could retrace interactions between (related or unrelated) components. This theoretical shift from a Tree of Life to a Dynamic Interaction Network of Life, which is supported by diverse molecular, cellular, microbiological, organismal, ecological and evolutionary studies, would further unify evolutionary biology.

## Deciphering diversity through evolution

The living world is nested and multilevel, involves multiple agents and changes at different timescales. Evolutionary biology tries to characterize the dynamics responsible for such complexity to decipher the processes accounting for the past and extant diversity observed in molecules (namely, genes, RNA, proteins), cellular machineries, unicellular and multi-cellular organisms, species, communities and ecosystems. In the 1930s and 1940s, a unified framework to handle this task was built under the name of Modern Synthesis [1]. It encompassed Darwin’s idea of evolution by natural selection as an explanation for diversity and adaptation and Mendel’s idea of particular inheritance, giving rise to population and quantitative genetics, a theoretical frame that corroborated Darwin’s hypothesis of the paramount power of selection for driving adaptive evolution [2]. This framework progressively aggregated multiple disciplines: behavioural ecology, microbiology, paleobiology, etc. Overall, this classic framework considers that the principal agency of evolution is natural selection of favourable variations, and that those variations are constituted by random mutations and recombination in a Mendelian population. The processes of microevolution, modelled by population and quantitative genetics, are likely to be extrapolated to macroevolution [3]. To this extent, models that focus on one or two loci are able to capture much of the evolutionary dynamics of an organism, even though in reality many interdependencies between thousands of loci (epistasis, dominance, etc.) occur as the basis of the production and functioning of a phenotypic trait. Among forces acting on populations and modelled by population geneticists, natural selection is the one that shapes traits as adaptations and the design of organisms; adaptive radiation then explains much of the diversity; and common descent from adapted organisms explains most of the commonalities across living forms (labelled homologies), and allows for classifying living beings into phylogenetic trees. Evolution is gradual because the effects of mutations are generally small, large ones being most likely to be deleterious as theorized by Fisher’s geometric model [4].

Many theoretical divergences surround this core view: not everyone agrees that evolution is change in allele frequencies, or that population genetics captures the whole of the evolutionary process, or that the genotypic viewpoint — tracking the dynamics of genes as ‘replicators’ [5] or the strategy ‘choices’ of organisms as fitness maximizing agents [6] — should be favoured to understand evolution. Nevertheless, it has been a powerful enough framework to drive successful research programs on speciation, adaptation, phylogenies, evolution of sex, cooperation altruism, mutualism, etc., and incorporate apparent challenges such as neutral evolution [7], acknowledgement of constraints on variation [8], or the recent theoretical turn from genetics to genomics following the achievement of the Human Genome Program [9]. Causation is here overall conceived of as a linear causal relation of a twofold nature: from the genotype to the phenotype (assuming of course environmental parameters), and from the environment to the shaping of organisms via natural selection. For instance, in the classic case of evolution of peppered moths in urban forests at the time of the industrial revolution, trees became darkened with soot, and then natural selection favored darker morphs as ‘fitter’ ones, due to their being less easily detected by predator birds, resulting in a relative increase in frequency of the darker morphs in the population [10].

Yet in the last 15 years biologists and philosophers of biology have regularly questioned the genuinely unifying character of this Synthesis, as well as its explanatory accuracy [11]. Those criticisms questioned notably the set of objects privileged by the Modern Synthesis, arguably too gene-centered [12], and its key explanatory processes, since niche construction [13], lateral gene transfer [14, 15], phenotypic plasticity [16, 17], and mass extinction [18] could, for example, be added [11]. Usually these critiques emphasize aspects rooted in a particular biological discipline: lateral gene transfer from microbiology, plasticity from developmental biology, mass extinction from paleobiology, ecosystem engineering from functional ecology, etc. There were also recurring claims for novel transdisciplinary fields: evo-eco-devo [19], investigating the evolutionary dynamics of host and microbe associations (forming combinations often referred to as holobionts), evolutionary cell biology [20], or microbial endocrinology [21], among others. This latter discipline aims at understanding the evolved interactions between microbial signals and host development. Indeed, it is compelling for evolutionary biologists to decipher how such multi-species interactions became established (namely, whether they involved specific microbial species and molecules, and whether they evolved independently in different host lineages).

Evolutionary biology is thus currently undergoing various theoretical debates concerning the proper frame to formulate it [11, 22–24]. Here, we introduce an original solution which moves this debate forward, acknowledging that nothing on Earth evolves and makes sense in isolation, thereby challenging the key assumption of the Modern Synthesis framework that targeting the individual gene or organism (even when in principle knowing that it is part of a set of complex interactions) allows us to capture evolution in all its dimensions. Since the living world evolves as a dynamic network of interactions, we argue that evolutionary biology could become a science of evolving networks, which would allow biologists to explain organisational complexity, while providing a novel way to reframe and to unify evolutionary biology.

## Biology is regulated by networks

### Networks at the molecular level

Although numerous studies have focused on the functions of individual genes, proteins and other molecules, it is increasingly clear that each of these functions belongs to complex networks of interactions. Starting at the molecular scale, the importance of a diversity of molecular agents, such as (DNA-based) genes and their regulatory sequences, RNAs and proteins, is well recognized. Importantly, in terms of their origins and modes of evolution, these agents are diverse. Genes are replicated across generations, via the recruitment of bases along a DNA template, thereby forming continuous lineages, affected by Darwinian evolution. By contrast, proteins are reconstructed by recruitment of amino acids at the ribosomal machinery. There is no physical continuity between generations of proteins, and thus no possibility for these agents to directly accumulate beneficial mutations [25]. Moreover, all these molecular entities are compositionally complex, in the sense that they are made of inherited or reassembled parts. *E pluribus unum*: genes and proteins are (often) conglomerates of exons, introns [26–28], and domains [29–31]. Similar claims can be made about composite molecular systems, such as CRISPR and Casposons [32, 33], etc. This modular organisation has numerous consequences: among them, genes can be nested within genes [34]; proteins congregate in larger complexes [35]. Importantly, this modularity is not the mere result of a divergence from a single ancestral form, but also involves combinatorial processes and molecular tinkering of available genetic material [36–38]. The coupling and decoupling of molecular components can operate randomly, as in cases of presuppression proposed to neutrally lead to large molecular complexes [39–41]. Presuppression, also known as constructive neutralism, is a process that generates complexity by mechanically increasing dependencies between interacting molecules, in the absence of positive selection. When a deleterious mutation affects one molecular partner, existing properties of another molecule with which the mutated molecule already interacted can compensate for its partner defect. Presuppression operates like a ratchet, since the likelihood to restore the original independency between molecules (by reverting the deleterious mutation) is lower than the likelihood to move away from this original state (by accumulating other mutations). Molecular associations can also evolve under constraints [42], eventually reinforcing the relationships between molecular partners, as suggested for some operons [43] and fused genes [44, 45].

Consistently, interconnectedness is a striking feature of the molecular world [46, 47]. Genes belong to regulatory networks with feedback loops [48]. Proteins belong to protein–protein interaction networks. This systemic view contrasts with former atomistic views assigning one function to one gene. First, it is not always correct that a gene produces only a protein, in the case of alternative splicing. Second, it is also unlikely that a protein performs one function, because no protein acts alone. Rather, biological traits result from co-production processes. This is nicely illustrated by the actual process of translation, during which both proteins and DNA necessarily interact, allowing for the collective reproduction of these two types of molecular agents. How these different components became so tightly integrated is a central issue for explaining evolution. Understanding how the molecular world functions and evolves therefore requires analysing molecular organisation and the evolution of the architecture of interaction networks, especially since this structure can partly explain molecular reactions [46, 47, 49, 50]. Thus, systems biologists search for common motifs in molecular interaction networks from different organisms, such as feed-forward loops, assuming that some of these recurring patterns, because they affect different gene or protein sets, may reflect general rules and constraints affecting the construction and evolution of biological organisations [46].

Focusing evolutionary explanations on the structure of the interactions between genes rather than on the primary sequence of the genes is fundamentally different from sequencing genes and inferring history from their sequences alone. One could think here of the case of explaining gene activation/repression. Comparative works on molecular interaction networks show that interactions affect the evolution of the molecules composing networks, which means that beyond compositional complexity, organisational complexity must be modeled to understand biological evolution [46, 51–54]. Before the analysis of complex networks, compensatory sets of elements, such as groups of sub-functional paralogous genes [55], or groups of genes with pressupressed mutations [39, 40], already stressed the evolutionary interdependence of molecules. However, compensatory interactions between agents, each of them being by themselves poorly adapted, ran counter to the intuition that natural selection will eliminate dysfunctional individual entities. Their recognition invites one to consider Earth as possibly populated by unions of individually dysfunctional agents rather than by the fittest survivors within individual lineages, possibly since early life, according to Woese’s theory on progenotes, namely communities of interacting protocells unable to sustain themselves alone, evolving via massive lateral genetic exchanges [56].

At the molecular level, it is reasonable to assume that processes resulting from interactions of a diversity of intertwined agents offer a crucial *explanans* of biological complexity. Rather than ‘one agent, one action’, it would be more accurate to consider ‘a relationship between agents, one action’ as the *modus operandi* of life. Multiple drivers, of different nature, contribute to the evolution of these interactions: among others, gene co-expression/co-regulation [57], sometimes mediated by transposons [58–61]; the evolutionary origin of the genes [62]; and also physical and chemical laws, as well as the presence of targeting machineries that constrain and regulate diffusion processes in the cell. These types of relationships described at the molecular level are also recovered at other levels of biological organisations.

### Networks at the cellular level

**Fig. 1.**
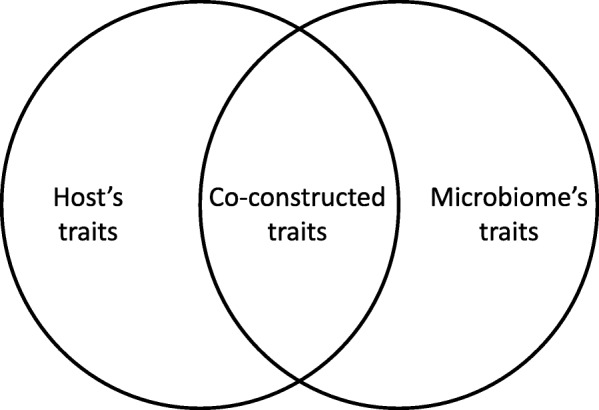
An example of co-construction, the case of holobionts. The *left circle* represents the set of traits associated with a host, the *right circle* represents the set of traits associated with its microbial communities; the intersected area represents traits that are produced jointly as a result of the interaction between hosts and microbes. When this area becomes large or when co-constructed traits are remarkable, they cannot be correctly explained under a simple model treating hosts and microbes in isolation. This scheme holds for different types of partners

Similar conclusions have been reached at the cellular level, also crucial for understanding life history. All prokaryotes and protists are unicellular organisations, and the cell is a fundamental building block of multicellular organisms. Cells must constantly evaluate the states of their inner and outer environments, i.e. to adjust their gene expression and react accordingly [46]. This involves regulatory, transduction, developmental, and protein interaction networks, etc. Cells are built upon inner networks of interacting components, and involved in or affected by a diversity of exchanges, influences and modes of communications (namely, genetic, energetic, chemical and electrical modes). Microbiology has gone a long way toward unraveling these processes since its heyday of pure culture studies, a fruitful reductionist approach now complemented by environmental studies. These latter further unraveled that cells compete and cooperate with, and even compensate for each other, within mono- or multispecific microbiomes [63, 64]. Both types of microbiomes have a fundamental commonality: they produce collective properties and co-constructed phenotypes (Fig. 1) evolving at the interface between cells. Such properties cannot be understood without considering networks of influences: the oscillatory growth of biofilms of *Bacillus subtilis* cannot be deduced from the analyses of the complete genomes of these clones, but requires modeling metabolic co-dependence within a monogenic community affected by a delayed feedback loop, involving chemical and electrical signals [65, 66].

Furthermore, many cellular agents show a relative lack of autonomy. In nature, some groups of prokaryotes display complementary genomes with incomplete metabolic pathways, consistent with the black queen hypothesis, which predicts that our planet is populated by groups of (inter)dependent microbes [67, 68]. More precisely, this hypothesis predicts the loss of a costly function, encoded by a gene or a set of genes, in individuals, when this function becomes dispensable at the individual level, since it is achieved by other individuals that produce (usually leaky) public goods in sufficient amount to support the equilibrium of the community. Thus, gene losses in some cells are compensated by leaks of substrates from other cells, formerly encoded by the lost genes. Some microbes experience labor division [69]. Symbionts and endosymbionts depend on their hosts. The ‘kill the winner’ theory [70] further challenges the notion that the microbial world is a world of fit cellular individuals. This theory stresses a collective process via which viruses mechanically mostly attack cells that reproduce faster and thus regulate bacterial populations, these latter sustaining their diversity because these populations are comprised of individual prokaryotic cells that make a suboptimal use of a diversity of resources. Thus, cells belong to networks that affect their growth and survival, which might explain why most bacteria cannot be grown in pure culture. They only truly thrive within communities, whose global genetic instructions are spread over several genetically incomplete microbes.

Accounting for these internal and external cellular networks requires considering processes that are not central in the synthetic evolutionary theory. Typically, the notion that cellular evolution makes jumps, because new components and processes (such as metabolic pathways) are acquired from outside a given cellular lineage, contrasts with more gradual accounts of biological change, like accounts based on point mutations affecting genes already present in the lineage. Because saltations (macromutations) are essential evolutionary outcomes of introgressive processes, via the combination of components from different lineages, no complete picture of evolution can be provided without these jumps, which are naturally modeled by networks. Indeed, genetic information has been flowing both vertically and horizontally between prokaryotes for over 3.5 billion years [71–77], and possibly earlier, according to Woese, who proposed that our universal ancestor was not an entity but a process, that is, genetic and energetic exchanges within protocellular communities [56]. Remarkably, this latter case indicates that network modeling could help to tackle a fundamental issue in evolutionary biology: modeling the evolution of biological processes that emerge from interactions between biological entities. Since these interactions can be represented by a network, the evolution of these interactions, describing the evolution of biological processes, can then be represented by dynamic networks. Likewise, eukaryogenesis rested on the co-construction of a novel type of cell, as a result of the endosymbiosis of a bacteria within an archaeon [78–80]. Later, the evolution of photosynthetic protists emerged from endosymbioses involving unicellular eukaryotes and cyanobacteria, or various lineages of protists, namely in secondary and tertiary endosymbioses [81]. Such endosymbioses, and their outcomes as illustrated in our work [82, 83], are also naturally modeled using networks.

Moreover, the long-term impact of these introgressive processes on cellular evolution should not be underestimated. For instance, endosymbiosis does not merely introduce new cellular lineages, it also favors the evolution of chimeric structures and chimeric processes within cells [83–91]. Such intertwining cannot be modeled using a single genealogical tree, which only recapitulates cellular divergence from a last common ancestor. Even though cells always derive from other cells, a full cellular history cannot be reduced to the history of some cellular components that are assumed to track the history of cellular division [92]. In particular, phylogenetic analyses of informational genes cannot be the only clue to understanding the origins of cellular diversity, since these genes do not reflect how cells are organized, how they gather their energy, and how they interact with each other. Analyzing the co-construction side of evolution requires enhanced models: understanding eukaryotic evolution requires mixed considerations of cellular architecture, population genetics and energetics, which go beyond classic phylogenetic models, which not so long ago were still prone to considering three primary domains of life [93–95].

Although invoking multiple agents rather than a single ancestor in evolutionary explanations might appear to contradict the famous Ockham’s razor [96], it does so only superficially when it is likely that many cells are co-constructed, especially in the context of a web of life. Enhanced models including intra- and extracellular interactions appear necessary to understand cellular complexity, including the predictable disappearance of traits (and processes), namely the convergent gene loss of mitochondria and plastids [97] by a process called dedarwinification [98, 99].

### Networks beyond the cellular level

Studies of multicellular organisms—we will focus on animals—have led to similar general findings. Understanding animal traits and their evolution requires analyzing the relationships between a multiplicity of agents belonging to different levels of biological organisation, eventually nested, some of which co-constructs animals and guarantees their complete lifecycle [100]. Because no sterile organism lives on Earth, animal development, health and survival depend on microbes. Granted, bacteria can often legitimately be seen as part of the environmental demands in an evolutionary model focused on the host’s lineage; or sometimes bacteria and host could also be considered as part of a coevolution process, with no need to posit the whole as a unit of selection [101]. However, asking ‘who is the beneficiary of the symbiosis as the result of evolution?’ may in some cases lead to the recognition that bacteria and host evolved together and were selected together [102]. More generally, while some microbes contribute to animals’ lives possibly as a result of host-derived selection, others contribute as a result of selectively neutral processes (like microbial priming [103]) [101, 104]. These interactions produce communication networks within the animal body: chemical information circulates between the animal brain and the gut microbiome. These interactions also result in communication and interaction networks between individuals. In some animal lineages, the microbiome affects social behaviors, for instance fermenting microbes inform about the gender and reproductive status in hyena [105]. Components of the microbiome also affect mating choice [106], reproductive isolation and possibly speciation. Consequently, the microbiome now appears as an essential component of animal studies [107]. Microbiome studies, the significance of which is overstated in some respects, nevertheless have shown that the evolutionary intertwining between many metazoa and commensal or symbiotic bacteria could not be neglected anymore and black-boxed in favor of purely host gene-centered evolutionary models. And the associations between hosts and microbes do not need to be units of selection to be part of the recent insights that support the novel theoretical framework proposed here. Their interplay imposes reconfigurations of practices, theories and disciplines [108]. As a result of our improved insight into evolution, zoology and immunology [109] become theaters of new ecological considerations [110], sometimes strangely qualified as Lamarckian [111, 112], because animals can recruit environmental microbes and transmit them (with a non-null heritability [113]) to their progeny. Therefore, nuclear gene inheritance alone may provide too narrow a perspective to account for the evolution of all animal traits; as an example, aphid body color depends on animal genetics and the presence of *Rickettsiella* [114]. Population genetics gets included in a broader community genetics, which also considers transmission of microbes and their genes [108, 114]. The use of gnotobiotic and transbiotic animals becomes a new experimental standard to analyze multigenomic collectives without counterparts in modern synthesis theories. These collectives harbor morphological, physiological, developmental, ecological, behavioral and evolutionary features [115–119] that are not purely constructed by animal genes, but rather appear to be co-constructed at the genetic and metabolic interface between the microbial and macrobial worlds, while the content of the respective animal genomes only provides incomplete instructions. Understanding animal evolution requires understanding the interaction networks between components from which these taxa evolved, and the networks to which these taxa still belong.

In ecology, an analogous turn towards network thinking has been promoted since the 1990s with the general acceptance of the notions of metapopulations [120] and then metacommunities [121]. These views suggest that the dynamics of ecological biodiversity is not so much located within a community of species but rather in a metacommunity, which can be thought of as a network of communities exchanging species, while targeting one community blinds one to what genuinely accounts for biodiversity and ecosystem functioning [122].

This quick overview provides evidence that networks are at the origin of the genes of unicellular and multicellular organisms and central for their functions. The living world is a world of ‘and’ and ‘co-’. From division of labor and compensations, to dependencies and co-constructions, etc.: interactions (which only begin to be deciphered) are everywhere in biology. Thus, explaining the actual features of biodiversity requires explaining how multiple processes, interface phenomena (co-construction of biological features, niche construction, metabolic cooperation, co-infection and co-evolution) and organisations (for instance, from molecular pathways to organisms and ecosystems) arose from interacting components, and how these processes, phenomena and organisations may have been sustained and transformed on Earth.

## Reframing evolutionary explanations from the scaffolded evolution perspective

### Introducing a classification of interacting components

While classic evolutionary models, prompted by Darwin’s famous tree [123], mostly stress how related entities diverge in relative independence, it appears important to show how a diversity of components, which may not be related, interact and produce various evolutionary patterns.

**Fig. 2.**
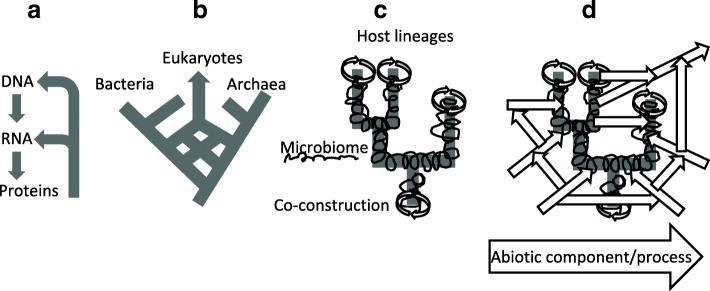
Different types of scaffolding, at four levels of biological organisations. **a** Functional interactions at the molecular level. **b** Introgression and vertical descent at the cellular level. **c** Co-construction at the multicellular level. **d** Niche-construction and physico-chemical interactions at the eco-systemic level

The notion of scaffolding [124], which describes how one entity continues an event initiated by another entity, and relies on it up to the point that at some timescale it becomes dependent upon it for further evolution, appears as a fundamental relationship to describe the evolution of life. We propose scaffolding should become more central in explanations of evolution because no components from the biological world are actually able to reproduce, or persist, alone (Fig. 2). Each entity influences or is influenced by something external to it, and is consequently part of a process. Scaffolding thus defines the causal backbone of collective evolution. It describes the historical continuity between temporal slices of interaction networks, since any evolutionary stage relies on previously achieved networks and organisations. Therefore, describing the evolution of interactions requires explanations to address the following issues: what scaffolds what, what transforms the environment of what, and are these influences reciprocal? Characterizing the types of components that, together, have evolutionary importance through their potential interaction is therefore a central step to expanding evolutionary theory.

**Fig. 3.**
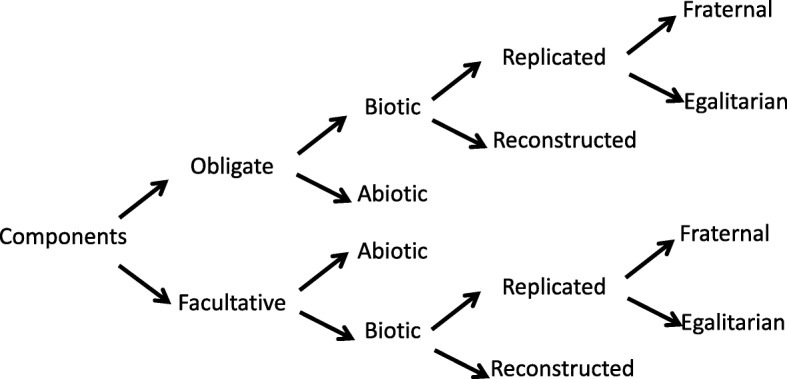
Classification of major types of components in evolving systems. A process/collective cannot be completed in the absence of obligate components, whereas facultative components do not affect the outcome of the process/function of the collective. Biotic components are biological, material products, whereas abiotic components are environmental, geological, chemical, physical or cultural artefacts. Replicated components are produced by replication, which implies a physical continuity between ancestral and descendent components; they undergo a paradigmatic Darwinian evolution. Reconstructed components are reproduced without direct physical continuity, and cannot directly accumulate beneficial mutations. Fraternal components belong to the same lineage, whereas egalitarian components belong to different lineages

We propose that a first distinction can be made between obligate and facultative components. Suppressing the former impacts the course and eventually the reproduction of the process to which they contribute (Fig. 3), whereas facultative components do not hold such a crucial role, and may simply be involved by chance. A second distinction is whether the components are biotic (genes, proteins, organisms…) or abiotic (such as minerals, environmental, cultural artefacts). Abiotic components can be recruited from the environment or be shaped by biological processes [125]. They can also alter the evolution of the biotic components, for example, environmental change can drive genetic and organismal evolution and selection. The history of life clearly depends on the interplay of both types of components. Biotic components, however, deserve a specific focus. Some of them form lineages (for instance, genes replicate), while others do not (for instance, proteins are reconstructed). Finally, interacting replicated components can be further classified into fraternal components when they share a close last common ancestor (e.g. in kin selection cases), and egalitarian components, when they belong to distinct lineages (as an example, think of the evolution of chimeric genes by fusion and shuffling [29, 45, 126]) [63].

### Introducing dynamic interaction networks

Biodiversity usually evolves from interactions between the diverse types of components described above. For example, metalloproteases emerge from the interaction between reconstructed biotic components (proteins) and a metal ion. Regulatory networks involve biotic components that can be either replicated (i.e. genes and promoters) or reconstructed (i.e. proteins). Protein interaction networks intertwine reconstructed egalitarian biotic components, which means proteins that are not homologous. Evolutionary transitions such as eukaryogenesis result from the interweaving of biotic components (cells) from multiple lineages. Holobionts evolve from interactions between egalitarian biotic components (macrobial hosts and microbial communities) and possibly abiotic components, such as the mineral termite mounds, or the volatile chemicals produced by the microbial communities of hyenas [105].

**Fig. 4.**
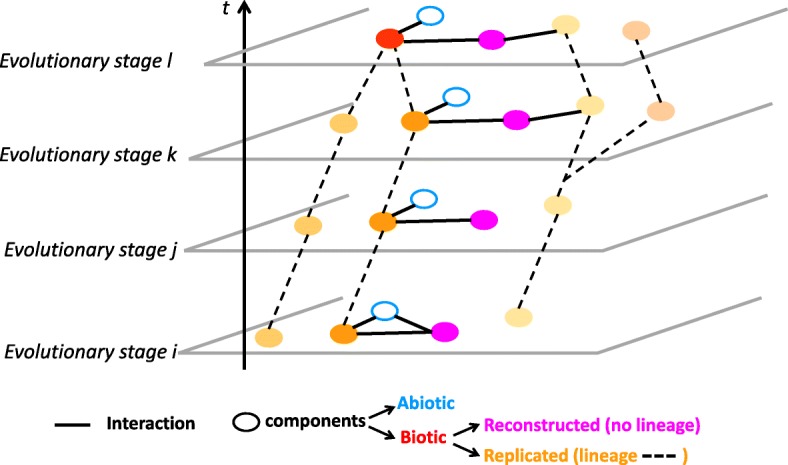
An evolving interaction network. Nodes are components (*circles* are full when the component is biotic). *Thick black edges* represent interactions between these components. The network topology evolves as nodes or their connection change. *Dashed edges* represent the phylogenetic ancestry of lineage-forming components

Taking collectives of interacting components as central objects of study in evolutionary biology invites us to expand the methods of this field. It encourages developing statistical approaches or inference methods beyond those operating under the very common assumption that biological components are independent. Therefore, we propose to represent interactions between components in the form of networks in which components are nodes and their interactions (of various sorts) are edges. These networks are conceptually simple objects. They can be described as adjacency lists of interactions, in the form ‘component A interacts with component B, at time *t* (when such a temporal precision is known)’. Such dynamic interaction networks could become more central representations and analytical frameworks, and serve as a common *explanans* for various disciplines in an expanded evolutionary theory. Importantly, because these networks embed both abiotic and biotic, related and unrelated components (like viruses, cells and rocks), they should not be conflated with phylogenetic networks, but recognized as a more inclusive object of study (Fig. 4). Where phylogenies describe relationships, networks can describe organisations. How such organisations evolve could for example be described by identifying evolutionary stages, that is, sets of components and of their interactions simultaneously present in the network (Fig. 4). Investigating the evolution of an ecosystem corresponds to studying the succession of evolutionary stages in such networks and detecting possible regularities—in the sense that some evolutionary stages would fully or partly reiterate over time—or hinting at rules or constraints (like architectural contingencies [127, 128] or principles of organisations [46]) on the recruitment, reproduction and heritability of their components.

Thus, we suggest that evolutionary biology could be reframed as a science of evolving networks, because such a shift would allow inclusive, multilevel studies of a larger body of biological and abiotic data, via approaches from network sciences.

## Concrete strategies to enhance network-based evolutionary analyses

Enhancing network-based evolutionary analyses, beyond the now classic research program of phylogenetic networks, could consolidate comparative analyses in the nascent field of evolutionary systems biology [129, 130], as illustrated by examples based on molecular networks. Network construction/gathering constitutes the first step of such analyses. This involves first defining nodes of the network, namely components suspected to be involved in a given system, and edges, namely qualitative (or quantitative, when weighted) interactions between these entities. Many biological interaction networks (gene co-expression networks (GCNs), gene regulatory networks (GRNs), metabolic networks, protein–protein interaction networks (PPIs), etc. [46]) are already known for some species, or can be inferred [131–136]. For example, GCNs offer an increasingly popular resource to study the evolution of biological pathways [137], as well as to reveal conservation and divergence in gene regulation [138]. GCNs are already used for micro-evolution studies, as in the case of fine-grained comparisons of expression variations between orthologous genes across closely related species, and for the analysis of minor evolutionary and ecological transitions, such as changes of ploidy [139, 140], adaptation to salty environments [141] or drugs [142], or the effects of plant domestication [143, 144]. Likewise, GRNs are starting to be used in micro-evolution and phenotypic plasticity studies [145]. Understanding the dynamics of GRNs appears critical to inferring the evolution of organismal traits, in particular during metazoan [146–148], plant [149] and fungal [150] evolution. We suggest that PPI, GCN and GRN studies could become mainstream and also be conducted at (much) larger evolutionary and temporal scales, to analyze additional, major, transitions.

**Fig. 5.**
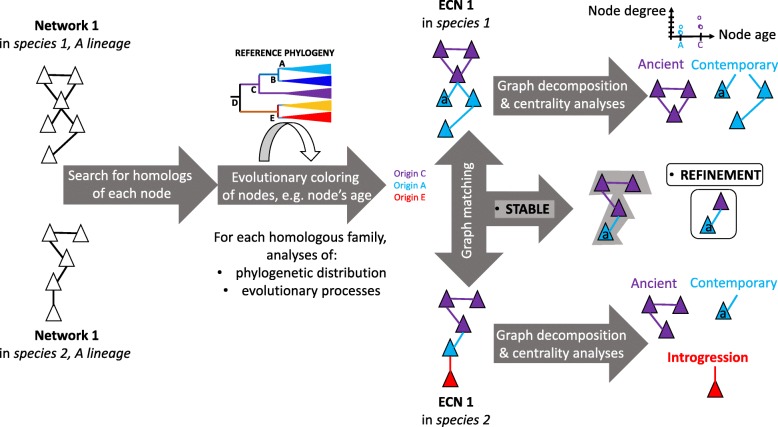
Workflow of the evolutionary analysis of interaction networks. From *left* to *right*: *triangles* represent components of interaction networks, *edges* between *triangles* represent interactions between these components. Interaction networks are first constructed/inferred, then their nodes and edges are colored to produce evolutionary colored networks (ECNs) that represent both the topological and the evolutionary properties of the networks. ECNs can be investigated individually by graph decomposition and centrality analyses, or several ECNs can be compared by graph alignment. The two types of comparisons can return conserved subgraphs that allow understanding of the dynamics of interaction networks, meaning when different sets of interactions (hence processes) evolved, and whether these interactions were evolutionarily stable. *Ancient* and *Contemporary* refer to the relative age of the sub-graphs, identifying new clade-specific relationships (here called refinement); introgression indicates that a component, and the relationship it entertains with the rest of the network, was inferred to result from a lateral acquisition

Based on these established networks, two major types of evolutionary analyses (network-decomposition and graph-matching; Fig. 5) can be easily further developed by evolutionary biologists. More precisely, the above-mentioned kinds of biological networks could be systematically turned into what we call evolutionary colored biological networks (ECNs). In ECNs, each node of a given biological network is colored to reflect one or several evolutionary properties. For example, in molecular networks, nodes correspond to molecular sequences (genes, RNA, proteins) that belong to homologous families that phylogenetic distribution across host species allows us to date [137, 151–156]. The ‘age’ of the family at the node can thus become one evolutionary color (Fig. 5). Likewise, several processes affecting the evolution of a molecular family (selection, duplication, transfer, and divergence in primary sequence) can be inferred by classic phylogenetic analyses or, as we proposed, by analyses of sequence similarity networks [157]. Such studies provide additional evolutionary colors (like quantitative measures: intensity of selection, rates of duplication, transfer, and percentage of divergence), which can be associated with nodes in ECNs [139, 149, 154, 158–161]. Thus, ECNs contain both topological information, characteristic of the biological network under investigation, as well as evolutionary information: what node belongs to a family prone to duplication, divergence, or lateral transfer, as well as when this family arose. Combining these two types of information in a single graph allows us to test specific hypotheses regarding evolution.

Using ECNs, it is first fruitful to test whether (or which of) these evolutionary colors correlates with topological properties of the ECNs [162–164]. The null hypothesis that nodes’ centrality, e.g. nodes’ positions in the network, is neither correlated with the age nor with the duplicability, transferability or divergence of the molecular entities represented by these nodes can be tested. Rejection of this hypothesis would hint at processes that affect the topology of biological networks or are affected by the network topology. For example, considering degree in networks, proteins with more neighbors are less easily transferred [163], highly expressed genes, more connected in GCNs, evolve slower than weakly expressed genes [165], and genes with lower degrees have higher duplicability in yeast, worm and flies [166]. Considering position in networks, node centrality correlates with evolutionary conservation [136], gene eccentricity correlates with level of gene expression and dispensability [167], and proteins interacting with the external environment have higher average duplicability than proteins localized within intracellular compartments [168]. Additionally, network structure gives a clue to evolution since old proteins have more interactions than new ones [169, 170]. Generalizing these disparate studies could help to understand the dynamics of biological networks, in other words how the architecture, the nodes and edges of present day networks, evolved and whether their changes involved random or biased sets of nodes and edges or follow general models of network growth with detectable drivers.

This focus would complement a classic tree-based view. For instance, under the reasonable working hypothesis that pairs of connected nodes of a given age reflect an interaction between nodes that may have arisen at that time [154, 171], ECNs can easily be easily decomposed into sub-networks, featuring processes of different ages (that is, sets of nodes of a given age, e.g. sets of interacting genes). This strategy allows identification of conserved network patterns, possibly under strong selective pressure [159]. Constructing and exploiting ECNs from bacteria, archaea, and eukaryotes thus has the potential to define conserved ancestral sets of relationships between components, allowing evolutionary biologists to infer aspects of the early biological networks of the last common ancestor of eukaryotes, archaea and bacteria and even of the last universal common ancestor of cells. Assuming that some of these topological units correspond to functional units [172], especially for broadly conserved subgraphs [138, 149, 152, 166, 173–182], would allow network decompositions to propose sets of important processes associated with the emergence of major lineages.

Moreover, graph-matching of ECNs allows several complementary analyses. First, for interaction networks, such as GRNs, whose sets of components and edges evolve rapidly [183–185], it becomes relevant to analyze where in the network such changes occur in addition to (simply) tracking conserved sets of components and edges. Whereas the latter can test to what extent conservation of the interaction networks across higher taxa supports generalizations made from a limited number of model species [186], the former allows us to test a general hypothesis: are there repeated types of network changes? For example, does network modification primarily affect nodes with particular centralities, as exemplified by terminal processes [187], or modules? Systematizing these analyses would provide new insights into whether the organisation principles of biological networks changed when major lineages evolved or remained conserved. In terms of the ECN, can the same model of graph evolution explain the topology of ECNs from different lineages? The null hypothesis would be that these major transitions left no common traces in biological networks. An alternative hypothesis would be that the biological networks convergently became more complex (more connected and larger) during these transitions to novel life forms. Indeed, analyses conducted on a few taxa have reported quantifiable and qualifiable modifications in biological networks (in response to environmental challenges [188], during ecological transitions [189] or as niche specific adaptations [190]). More systematic graph-matching [191–193] and motif analyses, comparing the topology of ECNs from multiple species, could likewise be used to test the hypothesis that major lineages are enriched in particular motifs (either modules of colored nodes and edges, or specific topological features, such as feed-forward loops [46] or bow-ties [194]). It would also allow identification of functionally equivalent components across species, namely different genes with similar neighbors in different species [176].

While inferences on conserved sets of nodes and edges in ECNs are likely to be robust (since the patterns are observed in multiple species), missing data (missing nodes and edges) constitute a recognized challenge, especially for the interpretation of what will appear in ECN studies as the most versatile (least conserved) parts of the biological networks. The issue of missing data, however, is not specific to network-based evolutionary analyses, and should be tackled, as with other comparative approaches, by the development and testing of imputation methods [195–197]. Moreover, issues of missing data can also be addressed by the production of high coverage -omics datasets in simple systems, allowing for (nearly) exhaustive representations of the entities and their interactions (i.e. PPIs, GCNs and GRNs within a cell, or metabolic networks within a species poor ecosystem). This kind of data would allow testing for the existence of selected emergent ecosystemic properties (like carbon fixation), as stated by the ITSNTS hypothesis [198]. For instance, deep coverage time series of metagenomic/metatranscriptomic data coupled with environmental measures from a simple microbial ecosystem, such as carbon fixation, could produce enough data to allow the evolutionary coloring of nodes of metabolic networks. Comparing ECNs representing, at each time point, the origin and abundance of the lineages hosting the enzymes involved in carbon fixation could test whether some combinations of lineages are repeated over time, and whether the components (e.g. genes and lineages) vary, whereas carbon fixation is maintained in the ecosystem, which would suggest that this process evolves irrespective of the nature of the interacting components.

Finally, entities from different levels of biological organisation (domains, genes, genomes, lineages, etc.) could also be studied together in a single network framework, by integrating them into multipartite networks [199]. Recently, our studies and others (see [200] and references therein) have demonstrated that various patterns in multipartite graphs can be used to detect and test combinatorial (introgressive) and gradual evolution (by vertical descent) affecting genes and genomes. Decomposing multipartite networks into twins and articulation points could for example then be used to represent and analyze the evolution of complex composite molecular systems, such as CRISPR, or the dynamics of invasions of hairpins in genomes [201].

## Further justifications for a shift toward network thinking

### Enlargement of evolutionary biology

Focusing evolutionary explanations and theories on collectives of interacting components, which may be under selection, facilitate selection, or condition arrangements through neutral processes [39, 40, 202], and representing these scaffolding relationships using networks with biotic and abiotic components and a diversity of edges representing a diversity of interaction types would be an enlargement. Enlargements, as expressing the need to consider structures that are more general than what already exists, have already occurred within evolutionary theory, when simplifications from population genetics were relaxed with respect to the original formalization in the Modern Synthesis [203], to account for within-genome interaction [9], gene–environment covariance [204], parental effects [205], and extended fitness though generations [206]. It also occurred when reticulations representing introgressions were added to the evolutionary tree.

Interestingly, replacing standard linear models in evolutionary theory with network approaches would transcend several traditional axes structuring the debates in evolutionary biology. For instance, scaffolded evolution, the idea that evolution relies on what came before, is orthogonal to the distinction between vertical and horizontal descent, since both tree-like and introgressive evolution are particular cases of scaffolding. Scaffolded evolution is also orthogonal to the distinction between gradual and saltational evolution. Likewise, scaffolded evolution is orthogonal to the debates between the actual role of adaptations vs neutral processes. Selection is a key mode of evolution of collectives but not the only one. The processes involved in the forming and evolution of collectives are not even restricted to the key processes of the Modern Synthesis (drift, selection, mutation and migration) but embrace interactions such as facilitation—namely antagonistic interactions between two species that allow a third species to prosper by restraining one of its predators or parasites [207], presuppression [39, 40], etc. Consequently, some evolutionary concepts may become more important than they currently are to explain evolution. For example, contingency, which means the dependence of an evolutionary chain of events upon an event that itself is contingent, in the sense that it can’t be understood as a selective response to environmental changes [18, 208, 209], is often associated with extraordinary events, like mass decimation. Contingency could come to be seen as a less extraordinary mode of evolution in the history of life, since the ordinary course of evolution might include many cases of contingent events, that is, associations of entities in a transient collective, including any scaffolds—associations that are not necessarily selective responses or the outcomes of processes modeled in population genetics.

Likewise, adopting a broader ontology could affect how evolutionary theorists think about evolution. Population thinking and tree-thinking came after essentialist conceptions of the living words, when populations and lineages were recognized as central objects of evolutionary studies [210]. A shift towards collectives and scaffolded evolution might encourage a similar development: the emergence of an openly pluralistic processual thinking, consistent with Carl Woese’s proposal to reformulate our view of evolution in terms of complex dynamic systems [211].

### Further unifying the evolutionary theory

Using a network-based approach to analyse dynamic systems also permits explanations that rely purely on statistical properties [212] or on topological or graph theoretical properties [213, 214] besides standard explanations devoted to unravelling mechanisms responsible for a phenomenon. Moreover, because of the inclusiveness of the network model, disciplines already recognized for their contribution to evolutionary theory (microbiology, ecology, cell biology, genetics, etc.) could become even more part of an interdisciplinary research program on evolution, effectively addressing current issues, consistent with the repeated calls for transdisciplinary collaborations [19–21, 215]. Disciplines that were not central in the Modern Synthesis—chemistry, physics, geology, oceanography, cybernetics or linguistics—could aggregate with evolutionary biology. Since a diversity of components gets connected by a diversity of edges in networks featuring collectives, as a result of a diversity of drivers, several explanatory strategies could be combined to analyze evolution. This extension to seemingly foreign fields makes sense when the components/processes studied by these other disciplines are evolutionarily or functionally related to biotic components and processes (either as putative ancestors of biological components and processes, like the use of a proton gradient in cells, which possibly derived from geological processes affecting early life [216], or as descendants of biological systems, e.g. technically synthesized life forms, which have a potential to alter the future course of standard biological evolution).

Remarkably, this mode of unification of diverse scientific disciplines would be original: the integration would not be a unification in the sense of logical positivism [217]—namely reducing a theory to a theory with more basic laws, or a theory with a larger extension. It would be a piecemeal [218] unification. Some aspects would be unified through a specific kind of graph modeling (because some interactions, namely mechanical, chemical, ecological ones, and a range of time scales are privileged in a set of theories), while other theories might be unified by other graph properties (like different types of edges and components). For example, the fermentation hypothesis for mammalian chemical communication could be analyzed in a multipartite network framework, which would involve nodes corresponding to individual mammals, nodes corresponding to microbes, and nodes corresponding to odorous metabolites. Nodes corresponding to mammals could either be colored to reflect an individual’s properties (its lineage, social position, gender, sexual availability), or these nodes could be connected by edges that reflect these shared properties, which defines a first host subnetwork. This host subnetwork can itself be further connected to a second subnetwork, namely the microbial subnetwork in which nodes representing microbes, colored by phylogenetic origins, could be connected to reflect microbial interactions (gene transfer, competition, metabolic cooperation, etc.). Connections between the host and microbial subnetworks could simply be made by drawing edges between nodes representing individual mammals hosting microbes, and nodes representing these microbes. Moreover, nodes representing mammals and nodes representing microbes could be connected to nodes representing odorous metabolites to show what odours are associated with what combinations of hosts and microbes. Elaborating this network in a piecemeal fashion would involve cooperation between chemists, microbiologists, zoologists and evolutionary biologists.

Of note, the use of integrated networks could pragmatically address a deep concern for evolutionary studies, by connecting phenomena that occur at different timescales: development and evolution [219] or ecology and evolution [220]. Considering transient collectives (thus processes) as stable entities at a given time-scale, when these collectives change much more slowly than the process in which they take part, amounts to a focus on interactions occurring at a given time scale by treating the slower dynamics as stable edges/nodes. Then, various parts of the networks embody distinct timescales, which may provide a new form of timescale integration, working out the merging of timescales from the viewpoint of the model, and with resources intrinsic to the model itself. The reason for this is that a node in an interaction network N*i*, describing processes relevant at a time scale *i*, can itself be seen as the outcome of another (embedded) interaction network N*j*, unfolding at a time scale *j*. This nestedness typically occurs when the node in N*i* represents a collective process, involving components that evolve sufficiently slowly with respect to the system considered at the time scale *i* to figure as an entity, a node in N*i*. In the case of a PPI network N*i*, each node conventionally represents a protein, but the evolution of each protein could be further analysed as the result of mutation, duplication, fusion and shuffling events affecting the gene family coding the proteins over time; for instance, each protein could thus be represented as the outcome of interaction between domains in a domain–domain interaction network N*j*. Considering these two time-scales, it becomes apparent that gene families enriched in exon shuffling events, a process directly analysable in N*j*, have a higher degree in PPI networks represented at the time-scale N*i* [221].

## Predictions: discovery of co-constructed phenotypes

What possible findings may result from this perspective shift? One can only speculate, but the nature of the potential discoveries is exciting. At the molecular level, the structure and composition of regulatory networks and protein interaction networks could be substantially enhanced to scaffolding elements. Currently, these networks represent interactions within a single individual/species. Yet, viruses are everywhere, viral genes and proteins clearly influence the networks of their hosts, and likely constitute an actual part of their evolution. Thus, virogenetics, a novel transdiscipline, may prosper in an expanded evolutionary theory to show how and to what extent viruses co-construct their hosts, including perhaps reproductive-viruses, allowing their hosts to complete their lifecycles. At the cellular level, new modes of communication [222, 223] could be discovered, as possible viral and microbial languages and communication networks in biofilms would exemplify. At the level of multicellular organisms and holobionts, ‘symbiotic codes’, guiding the preferential association between hosts and symbionts, could be identified. At the level of phyla, hidden evolutionary transitions may be unraveled. While secondary (and tertiary) acquisitions of plastids have been documented [81], it might be shown that mitochondria too have been so acquired in some eukaryotic lineages (alongside the plastid or independently). Secondarily acquired mitochondria may provide their new hosts with additional compartments, where chimeric proteomes could assemble [91, 224] and perform original physiological processes. At the ecosystemic level, evolving networks could be used to model the changes and stases of our planet, grounding biotic lineages and processes in their environment, while highlighting potential regularities in the organisations and dynamics of ecosystems. What affects the stability of what over the course of evolution could thus become a central theme of an expanded evolutionary theory.

## Concluding remarks and open questions

Interactions are not merely a part of biological history, they are what made this history. But evolutionary biologists have certainly not reconstructed the Dynamic Interaction Network of Life (DINol) yet. Undertaking this endeavor, however, would emphasize the importance of processes. Our ancestors were processes. Our descendants and those of other life forms will be processes too. Some one hundred and fifty years after *On the Origin of Species*, which started a great evolutionary inquiry, evolutionists should prepare to face a larger challenge: expanding evolutionary theory to study the evolution of processes. With the development of -omics and network sciences, the concepts, data and tools for this research program are increasingly available.

## References

[1] Huxley J (1942). Evolution: the modern synthesis.

[2] Gayon J. Darwinism's struggle for survival: heredity and the hypothesis of natural selection. Cambridge: Cambridge University Press; 1998.

[3] Simpson GG (1944). Tempo and mode in evolution.

[4] Martin G, Lenormand T (2008). The distribution of beneficial and fixed mutation fitness effects close to an optimum. Genetics.

[5] Dawkins R (1982). The extended phenotype.

[6] Grafen A (2002). A first formal link between the Price equation and an optimisation program. J Theor Biol.

[7] Kimura M (1983). The neutral theory of molecular evolution.

[8] Maynard Smith J, Burian R, Kauffman S, Alberch P, Campbell J, Goodwin B, et al. Developmental constraints and evolution. Q Rev Biol. 1985;60:265–87.

[9] Griffiths P, Stotz S (2013). Genetics and philosophy: an introduction.

[10] Kettlewell HDB (1955). Selection experiments on industrial melanism in the Lepidoptera. Heredity.

[11] Laland K, Uller T, Feldman M, Sterelny K, Muller GB, Moczek A (2014). Does evolutionary theory need a rethink?. Nature.

[12] Bateson P (2005). The return of the whole organism. J Biosci.

[13] Odling-Smee J, Laland K, Feldman M (2003). Niche construction: the neglected process in evolution.

[14] Doolittle WF, Bapteste E (2007). Pattern pluralism and the Tree of Life hypothesis. Proc Natl Acad Sci U S A.

[15] Sapp J (2009). The new foundations of evolution: on the Tree of Life.

[16] Walsh DM (2015). Organisms, agency, and evolution.

[17] West-Eberhard MJ (2003). Developmental plasticity and evolution.

[18] Gould SJ (1989). Wonderful life. The Burgess shale and the nature of history.

[19] Gilbert SF, Bosch TC, Ledon-Rettig C (2015). Eco-evo-devo: developmental symbiosis and developmental plasticity as evolutionary agents. Nat Rev Genet.

[20] Lynch M, Field MC, Goodson HV, Malik HS, Pereira-Leal JB, Roos DS (2014). Evolutionary cell biology: two origins, one objective. Proc Natl Acad Sci U S A.

[21] Lyte M (2011). Probiotics function mechanistically as delivery vehicles for neuroactive compounds: Microbial endocrinology in the design and use of probiotics. BioEssays.

[22] Huneman P, Walsh D (2017). Challenging the modern synthesis: Development, adaptation and inheritance.

[23] Pigliucci M, Müller G (2011). Evolution: the extended synthesis.

[24] Wray GA, Hoekstra HE, Futuyma DJ, Lenski RE, Mackay TFC, Schluter D (2014). Does evolutionary theory need a rethink? No, all is well. Nature.

[25] Eigen M, Schuster P (1977). The hypercycle. A principle of natural self-organization. Part A: Emergence of the hypercycle. Die Naturwissenschaften.

[26] Doolittle WF. Genes in pieces: Were they ever together? Nature. 1978;272:581–2.

[27] Gilbert W (1978). Why genes in pieces?. Nature.

[28] Irimia M, Roy SW. Origin of spliceosomal introns and alternative splicing. Cold Spring Harb Perspec Biol. 2014;6:a016071 10.1101/cshperspect.a016071 PMC4031966 24890509

[29] de Souza SJ (2012). Domain shuffling and the increasing complexity of biological networks. BioEssays.

[30] Marsh JA, Teichmann SA (2010). How do proteins gain new domains?. Genome Biol.

[31] Wang M, Caetano-Anolles G (2009). The evolutionary mechanics of domain organization in proteomes and the rise of modularity in the protein world. Structure.

[32] Koonin EV, Makarova KS (2017). Mobile genetic elements and evolution of CRISPR-Cas systems: all the way there and back. Genome Biol Evol..

[33] Krupovic M, Béguin P, Koonin EV (2017). Casposons: mobile genetic elements that gave rise to the CRISPR-Cas adaptation machinery. Curr Opin Microbiol.

[34] Assis R, Kondrashov AS, Koonin EV, Kondrashov FA (2008). Nested genes and increasing organizational complexity of metazoan genomes. Trends Genet.

[35] Lynch M (2013). Evolutionary diversification of the multimeric states of proteins. Proc Natl Acad Sci U S A.

[36] Duboule D, Wilkins AS (1998). The evolution of 'bricolage'. Trends Genet.

[37] Jacob F (1977). Evolution and tinkering. Science.

[38] Wilkins A (2007). Between "design" and "bricolage": genetic networks, levels of selection, and adaptive evolution. Proc Natl Acad Sci U S A.

[39] Doolittle WF, Lukes J, Archibald JM, Keeling PJ, Gray MW (2011). Comment on "Does constructive neutral evolution play an important role in the origin of cellular complexity?". BioEssays.

[40] Gray MW, Lukes J, Archibald JM, Keeling PJ, Doolittle WF (2010). Cell biology. Irremediable complexity?. Science.

[41] Lukes J, Archibald JM, Keeling PJ, Doolittle WF, Gray MW (2011). How a neutral evolutionary ratchet can build cellular complexity. IUBMB Life.

[42] Jain R, Rivera MC, Lake JA (1999). Horizontal gene transfer among genomes: the complexity hypothesis. Proc Natl Acad Sci U S A.

[43] Lawrence JG, Roth JR (1996). Selfish operons: horizontal transfer may drive the evolution of gene clusters. Genetics.

[44] Promponas VJ, Ouzounis CA, Iliopoulos I (2014). Experimental evidence validating the computational inference of functional associations from gene fusion events: a critical survey. Brief Bioinformatics.

[45] Tsoka S, Ouzounis CA (2000). Prediction of protein interactions: metabolic enzymes are frequently involved in gene fusion. Nat Genet.

[46] Alon U (2006). An introduction to systems biology: design principles of biological circuits.

[47] Milo R, Shen-Orr S, Itzkovitz S, Kashtan N, Chklovskii D, Alon U (2002). Network motifs: simple building blocks of complex networks. Science.

[48] Britten RJ, Davidson EH (1969). Gene regulation for higher cells: a theory. Science.

[49] Artzy-Randrup Y, Fleishman SJ, Ben-Tal N, Stone L (2004). Comment on "Network motifs: simple building blocks of complex networks" and "Superfamilies of evolved and designed networks". Science.

[50] Sorrells TR, Johnson AD (2015). Making sense of transcription networks. Cell.

[51] Carroll SB (2005). Evolution at two levels: on genes and form. PLoS Biol.

[52] Mallarino R, Grant PR, Grant BR, Herrel A, Kuo WP, Abzhanov A (2011). Two developmental modules establish 3D beak-shape variation in Darwin's finches. Proc Natl Acad Sci U S A.

[53] Peter IS, Davidson EH (2016). Implications of developmental gene regulatory networks inside and outside developmental biology. Curr Topics Dev Biol.

[54] Prud'homme B, Gompel N, Carroll SB (2007). Emerging principles of regulatory evolution. Proc Natl Acad Sci U S A.

[55] Force A, Lynch M, Pickett FB, Amores A, Yan YL, Postlethwait J (1999). Preservation of duplicate genes by complementary, degenerative mutations. Genetics.

[56] Woese C (1998). The universal ancestor. Proc Natl Acad Sci U S A.

[57] Coulombe-Huntington J, Xia Y (2017). Network centrality analysis in fungi reveals complex regulation of lost and gained genes. PLoS One.

[58] Chuong EB, Elde NC, Feschotte C (2017). Regulatory activities of transposable elements: from conflicts to benefits. Nat Rev Genet..

[59] Garcia-Perez JL, Widmann TJ, Adams IR (2016). The impact of transposable elements on mammalian development. Development.

[60] Imbeault M, Helleboid P-Y, Trono D (2017). KRAB zinc-finger proteins contribute to the evolution of gene regulatory networks. Nature.

[61] Sundaram V, Wang T (2018). Transposable element mediated innovation in gene regulatory landscapes of cells: re-visiting the "gene-battery" model. BioEssays.

[62] Ispolatov I, Yuryev A, Mazo I, Maslov S (2005). Binding properties and evolution of homodimers in protein-protein interaction networks. Nucleic Acids Res.

[63] Bapteste E (2014). The origins of microbial adaptations: how introgressive descent, egalitarian evolutionary transitions and expanded kin selection shape the network of life. Front Microbiol.

[64] Bapteste E, Lopez P, Bouchard F, Baquero F, McInerney JO, Burian RM (2012). Evolutionary analyses of non-genealogical bonds produced by introgressive descent. Proc Natl Acad Sci U S A.

[65] Liu J, Prindle A, Humphries J, Gabalda-Sagarra M, Asally M, Lee DY (2015). Metabolic co-dependence gives rise to collective oscillations within biofilms. Nature.

[66] Nunes-Alves C (2015). Biofilms: Electrifying long-range signalling. Nat Rev Microbiol..

[67] Morris JJ, Lenski RE, Zinser ER (2012). The Black Queen Hypothesis: evolution of dependencies through adaptive gene loss. MBio.

[68] Sachs JL, Hollowell AC (2012). The origins of cooperative bacterial communities. MBio.

[69] Bonner JT (1998). The origins of multicellularity. Integr Biol.

[70] Rodriguez-Valera F, Martin-Cuadrado AB, Rodriguez-Brito B, Pasic L, Thingstad TF, Rohwer F (2009). Explaining microbial population genomics through phage predation. Nat Rev Microbiol..

[71] Archibald JM (2015). One plus one equals one: symbiosis and the evolution of complex life. Eur J Phycol.

[72] Bapteste E, Anderson G. Intersecting processes are necessary explanans for evolutionary biology, but challenge retrodiction. In: Nicholson DJD, editor. Everything flows: Towards a processual philosophy of biology. Oxford: Oxford University Press. in press.

[73] Koonin EV (2015). The turbulent network dynamics of microbial evolution and the statistical Tree of Life. J Mol Evol.

[74] Lopez-Garcia P, Zivanovic Y, Deschamps P, Moreira D (2015). Bacterial gene import and mesophilic adaptation in archaea. Nat Rev Microbiol.

[75] Nelson-Sathi S, Dagan T, Landan G, Janssen A, Steel M, McInerney JO (2012). Acquisition of 1,000 eubacterial genes physiologically transformed a methanogen at the origin of Haloarchaea. Proc Natl Acad Sci U S A.

[76] Nelson-Sathi S, Sousa FL, Roettger M, Lozada-Chavez N, Thiergart T, Janssen A (2015). Origins of major archaeal clades correspond to gene acquisitions from bacteria. Nature.

[77] Levasseur A, Merhej V, Baptiste E, Sharma V, Pontarotti P, Raoult D (2017). The rhizome of Lokiarchaeota illustrates the mosaicity of archaeal genomes. Genome Biol Evol..

[78] Akanni WA, Siu-Ting K, Creevey CJ, McInerney JO, Wilkinson M, Foster PG (2015). Horizontal gene flow from Eubacteria to Archaebacteria and what it means for our understanding of eukaryogenesis. Philos Trans R Soc Lond Ser B Biol Sci.

[79] Martin WF, Garg S, Zimorski V (2015). Endosymbiotic theories for eukaryote origin. Philos Trans R Soc Lond Ser B Biol Sci.

[80] Pittis AA, Gabaldon T (2016). Late acquisition of mitochondria by a host with chimaeric prokaryotic ancestry. Nature.

[81] Archibald JM (2015). Genomic perspectives on the birth and spread of plastids. Proc Natl Acad Sci U S A.

[82] Méheust R, Bhattacharya D, Pathmanathan JS, McInerney JO, Lopez P, Bapteste E (2018). Formation of chimeric genes with essential functions at the origin of eukaryotes. BMC Biol.

[83] Meheust R, Zelzion E, Bhattacharya D, Lopez P, Bapteste E (2016). Protein networks identify novel symbiogenetic genes resulting from plastid endosymbiosis. Proc Natl Acad Sci U S A.

[84] Bailleul B, Berne N, Murik O, Petroutsos D, Prihoda J, Tanaka A (2015). Energetic coupling between plastids and mitochondria drives CO2 assimilation in diatoms. Nature.

[85] Bogumil D, Alvarez-Ponce D, Landan G, McInerney JO, Dagan T (2014). Integration of two ancestral chaperone systems into one: the evolution of eukaryotic molecular chaperones in light of eukaryogenesis. Mol Biol Evol.

[86] Dorrell RG, Howe CJ (2012). Functional remodeling of RNA processing in replacement chloroplasts by pathways retained from their predecessors. Proc Natl Acad Sci U S A.

[87] Gavelis GS, Hayakawa S, White RA, Gojobori T, Suttle CA, Keeling PJ (2015). Eye-like ocelloids are built from different endosymbiotically acquired components. Nature.

[88] Husnik F, Nikoh N, Koga R, Ross L, Duncan RP, Fujie M (2013). Horizontal gene transfer from diverse bacteria to an insect genome enables a tripartite nested mealybug symbiosis. Cell.

[89] Martin W, Koonin EV (2006). Introns and the origin of nucleus-cytosol compartmentalization. Nature.

[90] Nowack EC, Price DC, Bhattacharya D, Singer A, Melkonian M, Grossman AR (2016). Gene transfers from diverse bacteria compensate for reductive genome evolution in the chromatophore of Paulinella chromatophora. Proc Natl Acad Sci U S A.

[91] Stairs CW, Leger MM, Roger AJ (2015). Diversity and origins of anaerobic metabolism in mitochondria and related organelles. Philos Trans R Soc Lond Ser B Biol Sci.

[92] Bapteste E, O'Malley MA, Beiko RG, Ereshefsky M, Gogarten JP, Franklin-Hall L (2009). Prokaryotic evolution and the tree of life are two different things. Biol Direct.

[93] Koonin EV (2015). Energetics and population genetics at the root of eukaryotic cellular and genomic complexity. Proc Natl Acad Sci U S A.

[94] Martin W, Muller M (1998). The hydrogen hypothesis for the first eukaryote. Nature.

[95] Williams TA, Embley TM (2015). Changing ideas about eukaryotic origins. Philos Trans R Soc Lond Ser B Biol Sci.

[96] Simplicity BA. The Stanford Encyclopedia of Philosophy: Metaphysics Research Lab, Stanford University; 2016. https://plato.stanford.edu/archives/win2016/entries/simplicity/

[97] Allen JF (2015). Why chloroplasts and mitochondria retain their own genomes and genetic systems: Colocation for redox regulation of gene expression. Proc Natl Acad Sci U S A.

[98] Godfrey-Smith P (2015). Reproduction, symbiosis, and the eukaryotic cell. Proc Natl Acad Sci U S A.

[99] Karnkowska A, Vacek V, Zubacova Z, Treitli SC, Petrzelkova R, Eme L (2016). A eukaryote without a mitochondrial organelle. Curr Biol.

[100] Gilbert SF, Sapp J, Tauber AI (2012). A symbiotic view of life: we have never been individuals. Q Rev Biol.

[101] Moran NA, Sloan DB (2015). The hologenome concept: helpful or hollow?. PLoS Biol.

[102] Bouchard F (2009). Understanding colonial traits using symbiosis research and ecosystem ecology. Biol Theory..

[103] Selosse MA, Bessis A, Pozo MJ (2014). Microbial priming of plant and animal immunity: symbionts as developmental signals. Trends Microbiol.

[104] Theis KR, Dheilly NM, Klassen JL, Brucker RM, Baines JF, Bosch TC (2016). Getting the hologenome concept right: an eco-evolutionary framework for hosts and their microbiomes. mSystems.

[105] Theis KR, Venkataraman A, Dycus JA, Koonter KD, Schmitt-Matzen EN, Wagner AP (2013). Symbiotic bacteria appear to mediate hyena social odors. Proc Natl Acad Sci U S A.

[106] Sharon G, Segal D, Ringo JM, Hefetz A, Zilber-Rosenberg I, Rosenberg E (2010). Commensal bacteria play a role in mating preference of Drosophila melanogaster. Proc Natl Acad Sci U S A.

[107] Bosch TC, McFall-Ngai MJ (2011). Metaorganisms as the new frontier. Zoology.

[108] Bordenstein SR, Theis KR (2015). Host biology in light of the microbiome: ten principles of holobionts and hologenomes. PLoS Biol.

[109] Bosch TC (2014). Rethinking the role of immunity: lessons from Hydra. Trends Immunol.

[110] Costello EK, Stagaman K, Dethlefsen L, Bohannan BJ, Relman DA (2012). The application of ecological theory toward an understanding of the human microbiome. Science.

[111] Barr JJ, Auro R, Furlan M, Whiteson KL, Erb ML, Pogliano J (2013). Bacteriophage adhering to mucus provide a non-host-derived immunity. Proc Natl Acad Sci U S A.

[112] Rosenberg E, Sharon G, Zilber-Rosenberg I (2009). The hologenome theory of evolution contains Lamarckian aspects within a Darwinian framework. Environmental Microbiol.

[113] Ley RE (2015). The gene-microbe link. Nature.

[114] Tsuchida T, Koga R, Horikawa M, Tsunoda T, Maoka T, Matsumoto S (2010). Symbiotic bacterium modifies aphid body color. Science.

[115] Bravo JA, Forsythe P, Chew MV, Escaravage E, Savignac HM, Dinan TG (2011). Ingestion of Lactobacillus strain regulates emotional behavior and central GABA receptor expression in a mouse via the vagus nerve. Proc Natl Acad Sci U S A.

[116] Dupressoir A, Lavialle C, Heidmann T (2012). From ancestral infectious retroviruses to bona fide cellular genes: role of the captured syncytins in placentation. Placenta.

[117] Emera D, Casola C, Lynch VJ, Wildman DE, Agnew D, Wagner GP (2012). Convergent evolution of endometrial prolactin expression in primates, mice, and elephants through the independent recruitment of transposable elements. Mol Biol Evol.

[118] Ezenwa VO, Gerardo NM, Inouye DW, Medina M, Xavier JB (2012). Microbiology. Animal behavior and the microbiome. Science.

[119] Scarborough CL, Ferrari J, Godfray HC (2005). Aphid protected from pathogen by endosymbiont. Science.

[120] Hanski I (1999). Metapopulation ecology.

[121] Leibold MA, Holyoak M, Mouquet N, Amarasekare P, Chase JM, Hoopes MF (2004). The metacommunity concept: a framework for multi-scale community ecology. Ecol Lett.

[122] Ricklefs RE (2008). Disintegration of the ecological community. Am Nat.

[123] Darwin CA (1859). On the origin of species by means of natural selection.

[124] Caporael L, Griesemer J, Wimsatt W. Scaffolding in evolution, culture, and cognition. Cambridge: MIT Press; 2013.

[125] Laland K, Matthews B, Feldman MW (2016). An introduction to niche construction theory. Evol Ecol.

[126] Rogers RL, Hartl DL (2012). Chimeric genes as a source of rapid evolution in Drosophila melanogaster. Mol Biol Evol.

[127] Gould SJ, Lewontin RC (1979). The spandrels of San Marco and the Panglossian paradigm: a critique of the adaptationist programme. Proc R Soc Lond B Biol Sci.

[128] Sole RV, Valverde S (2006). Are network motifs the spandrels of cellular complexity?. Trends Ecol Evol.

[129] O'Malley MA, Soyer OS, Siegal ML (2015). A philosophical perspective on evolutionary systems biology. Biol Theory.

[130] Soyer OS, O'Malley MA (2013). Evolutionary systems biology: what it is and why it matters. BioEssays.

[131] Karimpour-Fard A, Hunter L, Gill RT (2007). Investigation of factors affecting prediction of protein-protein interaction networks by phylogenetic profiling. BMC Genomics.

[132] Koch C, Konieczka J, Delorey T, Lyons A, Socha A, Davis K (2017). Inference and Evolutionary Analysis of Genome-Scale Regulatory Networks in Large Phylogenies. Cell Syst.

[133] Shahdoust M, Pezeshk H, Mahjub H, Sadeghi M (2017). F-MAP: a Bayesian approach to infer the gene regulatory network using external hints. PLoS One.

[134] Simonsen M, Maetschke SR, Ragan MA (2012). Automatic selection of reference taxa for protein-protein interaction prediction with phylogenetic profiling. Bioinformatics.

[135] Spanier KI, Jansen M, Decaestecker E, Hulselmans G, Becker D, Colbourne JK (2017). Conserved transcription factors steer growth-related genomic programs in Daphnia. Genome Biol Evol..

[136] Wang P, Yu X, Lü J (2014). Identification and evolution of structurally dominant nodes in protein-protein interaction networks. IEEE Trans Biomed Circuits Syst.

[137] Ruprecht C, Vaid N, Proost S, Persson S, Mutwil M (2017). Beyond genomics: studying evolution with gene coexpression networks. Trends Plant Sci.

[138] Netotea S, Sundell D, Street NR, Hvidsten TR (2014). ComPlEx: conservation and divergence of co-expression networks in A. thaliana, Populus and O. sativa. BMC Genomics.

[139] Conant GC, Wolfe KH (2006). Functional partitioning of yeast co-expression networks after genome duplication. PLoS Biol.

[140] You Q, Xu W, Zhang K, Zhang L, Yi X, Yao D (2017). ccNET: Database of co-expression networks with functional modules for diploid and polyploid Gossypium. Nucleic Acids Res.

[141] Tang J, Lin J, Li H, Li X, Yang Q, Cheng Z-M (2016). Characterization of CIPK family in Asian Pear (Pyrus bretschneideri Rehd) and co-expression analysis related to salt and osmotic stress responses. Front Plant Sci.

[142] Siwo GH, Tan A, Button-Simons KA, Samarakoon U, Checkley LA, Pinapati RS (2015). Predicting functional and regulatory divergence of a drug resistance transporter gene in the human malaria parasite. BMC Genomics.

[143] Hu G, Hovav R, Grover CE, Faigenboim-Doron A, Kadmon N, Page JT (2016). Evolutionary conservation and divergence of gene coexpression networks in Gossypium (cotton) seeds. Genome Biol Evol.

[144] Lu X, Li Q-T, Xiong Q, Li W, Bi Y-D, Lai Y-C (2016). The transcriptomic signature of developing soybean seeds reveals the genetic basis of seed trait adaptation during domestication. Plant J.

[145] Zandveld J, van den Heuvel J, Mulder M, Brakefield PM, Kirkwood TBL, Shanley DP (2017). Pervasive gene expression responses to a fluctuating diet in Drosophila melanogaster: The importance of measuring multiple traits to decouple potential mediators of life span and reproduction. Evolution.

[146] Halfon MS (2017). Perspectives on gene regulatory network evolution. Trends Genet.

[147] Simakov O, Kawashima T (2017). Independent evolution of genomic characters during major metazoan transitions. Dev Biol.

[148] Wang P, Zhao D, Rockowitz S, Zheng D (2016). Divergence and rewiring of regulatory networks for neural development between human and other species. Neurogenesis (Austin).

[149] Masalia RR, Bewick AJ, Burke JM (2017). Connectivity in gene coexpression networks negatively correlates with rates of molecular evolution in flowering plants. PLoS One.

[150] Muñoz A, Santos Muñoz D, Zimin A, Yorke JA (2016). Evolution of transcriptional networks in yeast: alternative teams of transcriptional factors for different species. BMC Genomics.

[151] Kacharia FR, Millar JA, Raghavan R (2017). Emergence of New sRNAs in Enteric Bacteria is Associated with Low Expression and Rapid Evolution. J Mol Evol.

[152] Kim HS, Mittenthal JE, Caetano-Anollés G (2006). MANET: tracing evolution of protein architecture in metabolic networks. BMC Bioinformatics.

[153] Leyn SA, Suvorova IA, Kazakov AE, Ravcheev DA, Stepanova VV, Novichkov PS (2016). Comparative genomics and evolution of transcriptional regulons inProteobacteria. Microb Genom.

[154] Liang C, Luo J, Song D (2014). Network simulation reveals significant contribution of network motifs to the age-dependency of yeast protein-protein interaction networks. Mol BioSyst.

[155] Mustafin ZS, Lashin SA, Matushkin YG, Gunbin KV, Afonnikov DA (2017). Orthoscape: a cytoscape application for grouping and visualization KEGG based gene networks by taxonomy and homology principles. BMC Bioinformatics..

[156] Thompson JR, Erkenbrack EM, Hinman VF, McCauley BS, Petsios E, Bottjer DJ (2017). Paleogenomics of echinoids reveals an ancient origin for the double-negative specification of micromeres in sea urchins. Proc Natl Acad Sci U S A.

[157] Corel E, Lopez P, Méheust R, Bapteste E (2016). Network-thinking: graphs to analyze microbial complexity and evolution. Trends Microbiol.

[158] Friedlander T, Prizak R, Barton NH, Tkačik G (2017). Evolution of new regulatory functions on biophysically realistic fitness landscapes. Nat Commun.

[159] Gouy A, Daub JT, Excoffier L (2017). Detecting gene subnetworks under selection in biological pathways. Nucleic Acids Res.

[160] MacKintosh C, DEK F (2017). Recent advances in understanding the roles of whole genome duplications in evolution. F1000Res.

[161] Nguyen Ba AN, Strome B, Osman S, Legere E-A, Zarin T, Moses AM (2017). Parallel reorganization of protein function in the spindle checkpoint pathway through evolutionary paths in the fitness landscape that appear neutral in laboratory experiments. PLoS Genet.

[162] Alvarez-Ponce D, Feyertag F, Chakraborty S (2017). Position matters: network centrality considerably impacts rates of protein evolution in the human protein-protein interaction network. Genome Biol Evol..

[163] Cohen O, Gophna U, Pupko T (2011). The complexity hypothesis revisited: connectivity rather than function constitutes a barrier to horizontal gene transfer. Mol Biol Evol.

[164] Raymond J, Segrè D (2006). The effect of oxygen on biochemical networks and the evolution of complex life. Science.

[165] Wolf YI, Carmel L, Koonin EV (2006). Unifying measures of gene function and evolution. Proc Biol Sci.

[166] Hase T, Niimura Y, Tanaka H (2010). Difference in gene duplicability may explain the difference in overall structure of protein-protein interaction networks among eukaryotes. BMC Evol Biol.

[167] Xu K, Bezakova I, Bunimovich L, Yi SV (2011). Path lengths in protein-protein interaction networks and biological complexity. Proteomics.

[168] Prachumwat A, Li W-H (2006). Protein function, connectivity, and duplicability in yeast. Mol Biol Evol.

[169] Peterson GJ, Pressé S, Peterson KS, Dill KA (2012). Simulated evolution of protein-protein interaction networks with realistic topology. PLoS One.

[170] Pawlowski PH, Kaczanowski S, Zielenkiewicz P (2013). A kinetic model of the evolution of a protein interaction network. BMC Genomics.

[171] Ruprecht C, Proost S, Hernandez-Coronado M, Ortiz-Ramirez C, Lang D, Rensing SA (2017). Phylogenomic analysis of gene co-expression networks reveals the evolution of functional modules. Plant J.

[172] Zhao Y, Mooney SD (2012). Functional organization and its implication in evolution of the human protein-protein interaction network. BMC Genomics.

[173] Akinola RO, Mazandu GK, Mulder NJ (2016). A quantitative approach to analyzing genome reductive evolution using protein-protein interaction networks: a case study of Mycobacterium leprae. Front Genet.

[174] Briones-Moreno A, Hernández-García J, Vargas-Chávez C, Romero-Campero FJ, Romero JM, Valverde F (2017). Evolutionary analysis of DELLA-associated transcriptional networks. Front Plant Sci.

[175] Hahn MW, Kern AD (2005). Comparative genomics of centrality and essentiality in three eukaryotic protein-interaction networks. Mol Biol Evol.

[176] Hansen BO, Vaid N, Musialak-Lange M, Janowski M, Mutwil M (2014). Elucidating gene function and function evolution through comparison of co-expression networks of plants. Front Plant Sci.

[177] Kelliher CM, Leman AR, Sierra CS, Haase SB (2016). Investigating conservation of the cell-cycle-regulated transcriptional program in the fungal pathogen, Cryptococcus neoformans. PLoS Genet.

[178] Martinez-Pastor M, Tonner PD, Darnell CL, Schmid AK (2017). Transcriptional regulation in Archaea: from individual genes to global regulatory networks. Annu Rev Genet.

[179] Phan HTT, Sternberg MJE (2012). PINALOG: a novel approach to align protein interaction networks--implications for complex detection and function prediction. Bioinformatics.

[180] Romero-Campero FJ, Perez-Hurtado I, Lucas-Reina E, Romero JM, Valverde F (2016). ChlamyNET: a Chlamydomonas gene co-expression network reveals global properties of the transcriptome and the early setup of key co-expression patterns in the green lineage. BMC Genomics.

[181] Tamames J, Moya A, Valencia A (2007). Modular organization in the reductive evolution of protein-protein interaction networks. Genome Biol.

[182] Wang D, He F, Maslov S, Gerstein M (2016). DREISS: using state-space models to infer the dynamics of gene expression driven by external and internal regulatory networks. PLoS Comput Biol.

[183] Aguilera F, McDougall C, Degnan BM (2017). Co-option and de novo gene evolution underlie molluscan shell diversity. Mol Biol Evol.

[184] Auman T, Chipman AD (2017). The evolution of gene regulatory networks that define arthropod body plans. Integr Comp Biol.

[185] Mateos JL, Tilmes V, Madrigal P, Severing E, Richter R, Rijkenberg CWM (2017). Divergence of regulatory networks governed by the orthologous transcription factors FLC and PEP1 in Brassicaceae species. Proc Natl Acad Sci U S A.

[186] Renvoisé E, Kavanagh KD, Lazzari V, Häkkinen TJ, Rice R, Pantalacci S (2017). Mechanical constraint from growing jaw facilitates mammalian dental diversity. Proc Natl Acad Sci U S A.

[187] Cary GA, Cheatle Jarvela AM, Francolini RD, Hinman VF (2017). Genome-wide use of high- and low-affinity Tbrain transcription factor binding sites during echinoderm development. Proc Natl Acad Sci U S A.

[188] Noble R, Noble D (2017). Was the watchmaker blind? Or was she one-eyed?. Biology (Basel).

[189] Capela D, Marchetti M, Clérissi C, Perrier A, Guetta D, Gris C (2017). Recruitment of a lineage-specific virulence regulatory pathway promotes intracellular infection by a plant pathogen experimentally evolved into a legume symbiont. Mol Biol Evol.

[190] Orsini L, Brown JB, Shams Solari O, Li D, He S, Podicheti R (2018). Early transcriptional response pathways in Daphnia magna are coordinated in networks of crustacean-specific genes. Mol Ecol.

[191] Dohrmann J, Puchin J, Singh R (2015). Global multiple protein-protein interaction network alignment by combining pairwise network alignments. BMC Bioinformatics.

[192] Emmert-Streib F, Dehmer M, Shi Y (2016). Fifty years of graph matching, network alignment and network comparison. Inf Sci.

[193] Kelley BP, Yuan B, Lewitter F, Sharan R, Stockwell BR, Ideker T (2004). PathBLAST: a tool for alignment of protein interaction networks. Nucleic Acids Res.

[194] Ni B, Ghosh B, Paldy FS, Colin R, Heimerl T, Sourjik V (2017). Evolutionary remodeling of bacterial motility checkpoint control. Cell Rep.

[195] Huisman M, Alhajj R, Rokne J (2014). Imputation of missing network data: some simple procedures. Encyclopedia of Social Network Analysis and Mining.

[196] Ogundijo OE, Elmas A, Wang X (2016). Reverse engineering gene regulatory networks from measurement with missing values. EURASIP J Bioinform Syst Biol.

[197] Shao M, Zhou S, Guan J (2015). Revisiting topological properties and models of protein-protein interaction networks from the perspective of dataset evolution. IET Syst Biol.

[198] Doolittle WF, Inkpen SA (2018). Processes and patterns of interaction as units of selection: An introduction to ITSNTS thinking. Proc Natl Acad Sci U S A.

[199] Wong DCJ, Matus JT (2017). Constructing integrated networks for identifying new secondary metabolic pathway regulators in grapevine: recent applications and future opportunities. Front Plant Sci.

[200] Corel E, Méheust R, Watson AK, McInerney JO, Lopez P, Bapteste E (2017). Bipartite network analysis of gene sharings in the microbial world. Mol Biol Evol.

[201] Seligmann H, Raoult D (2016). Unifying view of stem–loop hairpin RNA as origin of current and ancient parasitic and non-parasitic RNAs, including in giant viruses. Curr Opin Microbiol.

[202] Stoltzfus A (2012). Constructive neutral evolution: exploring evolutionary theory's curious disconnect. Biol Direct.

[203] Gillespie JH (2004). Population genetics: A concise guide.

[204] Barker G, Desjardins E, Pearce T (2013). Entangled Life: Organism and Environment in the Biological and Social Sciences.

[205] Bonduriansky R (2012). Rethinking heredity, again. Trends Ecol Evol.

[206] Lehmann L (2008). The adaptive dynamics of niche constructing traits in spatially subdivided populations: evolving posthumous extended phenotypes. Evolution.

[207] Bruno JF, Stachowicz JJ, Bertness MD (2003). Inclusion of facilitation into ecological theory. Trends Ecol Evol.

[208] Beatty J (2006). Replaying life’s tape. J Philosophy.

[209] Turner D, Malaterre C, Braillard PA (2015). Historical contingency and the explanation of evolutionary trends. Biological explanation: An enquiry into the diversity of explanatory patterns in the life sciences.

[210] O'Hara RJ (1997). Population thinking and tree thinking in systematics. Zool Scr.

[211] Woese CR (2004). A new biology for a new century. Microbiol Mol Biol Rev.

[212] Lange M (2013). Really statistical explanations and genetic drift. Philosophy Sci.

[213] Huneman P. Diversifying the picture of explanations in biological sciences: Ways of combining topology with mechanisms. Synthese. 2018;195:115–46.

[214] Jones N (2014). Bowtie structures, pathway diagrams, and topological explanation. Erkenntnis.

[215] Lane N, Martin WF, Raven JA, Allen JF (2013). Energy, genes and evolution: introduction to an evolutionary synthesis. Philos Trans R Soc Lond Ser B Biol Sci.

[216] Sousa FL, Thiergart T, Landan G, Nelson-Sathi S, Pereira IA, Allen JF (2013). Early bioenergetic evolution. Philos Trans R Soc Lond Ser B Biol Sci.

[217] Nagel E (1961). The structure of science.

[218] Booth A, Mariscal C, Doolittle WF (2016). The Modern Synthesis in the light of microbial genomics. Annu Rev Microbiol.

[219] Gilbert SF, Opitz JM, Raff RA (1996). Resynthesizing evolutionary and developmental biology. Dev Biol.

[220] Kokko H, Chaturvedi A, Croll D, Fischer MC, Guillaume F, Karrenberg S (2017). Can evolution supply what ecology demands?. Trends Ecol Evol.

[221] Cancherini DV, França GS, de Souza SJ (2010). The role of exon shuffling in shaping protein-protein interaction networks. BMC Genomics.

[222] Amin SA, Hmelo LR, van Tol HM, Durham BP, Carlson LT, Heal KR (2015). Interaction and signalling between a cosmopolitan phytoplankton and associated bacteria. Nature.

[223] Erez Z, Steinberger-Levy I, Shamir M, Doron S, Stokar-Avihail A, Peleg Y (2017). Communication between viruses guides lysis-lysogeny decisions. Nature.

[224] Dorrell RG, Gile G, McCallum G, Meheust R, Bapteste EP, Klinger CM (2017). Chimeric origins of ochrophytes and haptophytes revealed through an ancient plastid proteome. elife.

